# Quality of Life, Disability Level, and Pain Intensity among Patients after Lumbar Disc Surgery: An Observational Three-Month Follow-Up Study

**DOI:** 10.3390/healthcare11243127

**Published:** 2023-12-08

**Authors:** Monika Michalak, Adam Druszcz, Maciej Miś, Małgorzata Paprocka-Borowicz, Joanna Rosińczuk

**Affiliations:** 1 Department of Nursing and Obstetrics, Wroclaw Medical University, 51-618 Wroclaw, Poland; monika.michalak@umw.edu.pl; 2 Department of Neurosurgery, Provincial Specialist Hospital in Legnica, 59-220 Legnica, Poland; dr.druszcz@gmail.com; 3 Department of Neurosurgery, Specialist Hospital in Walbrzych, 58-309 Walbrzych, Poland; mis.neurochirurg@gmail.com; 4 Department of Physiotherapy, Wroclaw Medical University, 55-355 Wroclaw, Poland; malgorzata.paprocka-borowicz@umw.edu.pl

**Keywords:** low back pain, intervertebral disc degeneration, lumbar disc surgery, quality of life, disability level, pain intensity, patient-reported outcome measures

## Abstract

The prevalence of intervertebral disc degeneration in the lumbar region resulting in low back pain is high. One of the treatment options is neurosurgery. Previous studies and systematic reviews demonstrate the need to identify factors that affect the health-related quality of life of patients undergoing surgery. This study aimed to analyze the sociodemographic and clinical factors that affect the quality of life of patients undergoing lumbar disc surgery. A group of 128 patients was assessed for eligibility and qualified by radiological examinations for lumbar disc surgery by a neurosurgeon in the outpatient clinic. Finally, 110 patients were studied and evaluated 24 h and 3 months after surgery. Health-related quality of life (36-Item Short Form Survey, SF-36), disability level (Oswestry Disability Index, ODI), and pain intensity (Visual Analogue Scale, VAS) were assessed. The mean pain intensity before surgery was 7.8 ± 2.3 pts and decreased significantly 24 h after surgery, with a mean score of 3.8 ± 2.4 pts (*p* = 0.0000). After three months, the increase in pain intensity was at 4.8 ± 2.4 pts, but the score was still significantly better than before surgery (*p* = 0.0024). The mean ODI score before surgery was 29.3 ± 8.4 pts (slight disability), and three months after surgery, there was an insignificant increase to a mean value of 31.5 ± 10.4 pts (*p* = 0.0834). There was a statistically significant increase in quality-of-life scores at three months after surgery in the following domains: physical functioning (8.7%; *p* = 0.0176), bodily pain (26.2%; *p* = 0.0000), vitality (5.8%; *p* = 0.0132) and mental health (6.2%; *p* = 0.0163), and a decrease in role limitations due to physical problems (3.8; *p* = 0.0013) and general health perception (6.7%; *p* = 0.0112). In conclusion, the surgical procedure plays an important role in improving the quality of life of patients operated on for intervertebral disc degeneration. It was effective in reducing the pain level, especially 24 h after surgery; however, surgery did not affect the disability level.

## 1. Introduction

The prevalence of intervertebral disc degeneration (IDD) in the lumbar region, resulting in low back pain (LBP), is high. According to available sources, it is as high as 63% or more [1,2]. Men are more likely to complain about pain in this area. The peak incidence varies. In women, it occurs in the fifth decade of life, and in men, in the fourth [3]. Depending on the world region, country, and environment, the global prevalence of lumbosacral back pain is 12–33% [4], while an average of 22–65% of the world’s population is affected per year [5].

In Poland, LBP is reported by 38.4% of women and 21.2% of men, cervical spine pain by 21% of women and 13% of men, respectively, and thoracic spine pain by 19% of women and 12.9% of men [3]. Chronic and recurrent back pain may affect as much as 60% of the population and is the most common reason for presenting to general practitioners (GPs) [6]. Unfortunately, knowledge of the epidemiology of back pain and associated lumbosacral pain is limited, partly due to methodological problems. Respondents often attribute back pain to spinal conditions, regardless of the correct diagnosis or the actual cause of the complaint. It was found that the most common non-spinal causes of LBP were diseases such as nephrolithiasis, endometriosis, tumors, fibromyalgia, mental disorders, and pregnancy [7].

IDD severely limits mobility and discourages people from physical activity due to its long-term course. Patients are forced to limit their social life and take long-term medication, mainly painkillers. Due to the pain, they give up their jobs and hobbies, staying in bed until the symptoms subside. In addition, it causes several additional discomforts in the form of increased nervous tension, lowered mood and depression, and, as a result, even drug dependence and, thus, reduced quality of life (QOL) [8,9].

In the conservative treatment of IDD, patients are recommended to stay in bed in a position that suits them and use pharmacotherapy. Conservative treatment should be comprehensive, which means the use of multidirectional treatment and various physiotherapeutic and pharmacological methods, depending on the disease period. The acute period usually lasts 2–3 days, and a supine or side-lying position with the legs bent at the knee and hip joints at 90° is usually used. In this position, lumbar lordosis and anterior pelvic tilts are reduced, and the hamstring muscles are relaxed [10,11]. Gradually, exercises to strengthen the paraspinal muscles, back muscles, gluteal and abdominal muscles are introduced. Physical therapy, McKenzie exercises, therapeutic massage, dry needling, and manual therapy are also used [12,13,14,15,16,17,18]. 

Physical therapy and kinesitherapy are complemented by pharmacological treatment. Non-steroidal anti-inflammatory drugs (NSAIDs), myorelaxants, sedatives, and antidepressants are mainly administered [19]. However, conservative treatment does not always have the desired effect. If the symptoms of the disease do not improve, then the physician suggests introducing surgical treatment. Failure to improve with conservative treatment and the patient’s deteriorating condition with worsening neurological deficits is the most common indication for neurosurgical treatment to decompress the nerve root or spinal cord by the degenerated intervertebral disc [20].

Undoubtedly, broad-based exercise rehabilitation improves patient-reported outcome measures (PROMs) effectively and safely. Psychosocial factors, as well as a person’s physical condition, were also shown to contribute to QOL in people with chronic LBP, as demonstrated in a selection of 21 publications [21]. Previous studies and systematic reviews illustrate the need to identify factors that affect the QOL of patients undergoing surgery. Therefore, the primary aim of this study was to analyze and evaluate the QOL of patients undergoing surgery for lumbar IDD. The primary objective was to investigate the sociodemographic and clinical factors that affect patients’ QOL. The secondary objective was to evaluate the effectiveness of surgery in reducing the intensity and severity of LBP. We assumed that surgery would be effective and lead to improvements in the studied outcomes: health-related quality of life, disability level, and pain intensity.

## 2. Materials and Methods

### 2.1. Study Design and Participants

The study was conducted among 128 patients (56 females, 54 males) aged 18 to 76 years undergoing treatment at the Department of Neurosurgery of the Marciniak Specialist Hospital in Wrocław (Poland). The patients were qualified for surgery by a neurosurgeon in the outpatient clinic (elective admissions) or by a neurosurgeon on duty (emergency admissions), taking into account the clinical status of the patients and the diagnostic radiological examinations performed (MRI or CT). 

### 2.2. Qualification Procedure

Patients undergoing surgery for the first time were qualified for the study group. Participation in the study was offered to all patients with lumbar disc disease (lumbar discopathy) who met the following inclusion criteria. Inclusion criteria: (1) age: 18 years and over, qualified for surgery for the first time, (2) diagnosed lumbar disc disease, (3) written informed consent to participate in the study, (4) participants registering for the second stage of the study. Criteria for exclusion from the study: (1) lack of consent for the study, (2) age below 18 years, (3) mental disorders, (4) failure of the participant to register for the second stage of the study, (5) other disorders that make it impossible to complete the questionnaires (e.g., cognitive disorders).

### 2.3. Data Collection

Contact details were also collected from the study participants, which, with their consent, would be used for contact after discharge. The final diagnosis and data on the surgical procedure performed were obtained from surgical protocols. A total of 128 patients were included in the study group. Three months after the day of surgery, an invitation to a follow-up study was sent to all patients in the study group. The participants again completed questionnaires assessing health-related quality of life (HRQOL) (36-Item Short Form Survey, SF-36), disability level (Oswestry Disability Index, ODI), and pain intensity (Visual Analogue Scale, VAS). Eighteen subjects did not participate in the follow-up study despite an invitation. Finally, a complete set of data obtained from 110 patients was analyzed. 

### 2.4. Outcome Measures

On admission to the Department, qualified patients were asked to complete a self-administered questionnaire regarding sociodemographic and clinical data and questionnaires related to PROMs: (1) an assessment of pain intensity based on the VAS before surgery, 24 h after surgery and three months after discharge from the Department; (2) a survey based on the Oswestry Low Back Pain Disability Questionnaire (also known as ODI) before surgery and three months after discharge from the Department; (3) a survey based on the Short Form Health Quality of Life Survey 36-Item (SF-36) before surgery and three months after discharge from the Department.

#### 2.4.1. Pain Intensity

The visual pain scale, VAS, is one of the most common and widely used measurement tools for determining pain intensity. Two ends were marked on a horizontal line of 100 mm: on one end was “no pain”, and on the other was “maximum pain the subject can imagine”. The patient indicated the pain level between these two ends by placing a vertical line. The following criteria were used for the assessment of pain: 0—no pain, 0.1–2—mild pain, 2.1–4—moderate pain, 4.1–6—moderate pain, 6.1–8—severe pain, and 8.1–10—very severe unbearable pain [22].

#### 2.4.2. Disability Level

The ODI makes it possible to assess the level of disability caused by LBP complaints. When completing the questionnaire, the patient answered ten questions about pain intensity, self-care (washing, dressing, etc.), lifting objects, walking, sitting, standing, sleeping, sex life (if applicable), social life, and traveling. A maximum of 5 points and a minimum of 0 points could be obtained for each question. The aggregate score is presented on a point scale from 0 to 50 points or on a percentage scale from 0 to 100%. The maximum score of the questionnaire could be 50 points (most severe patient condition), and the minimum score was 0 (no dysfunction). The lower the number of points calculated from the questionnaire, the better the patient’s health status. Based on the ODI, the patients were categorized according to their disability level. Disability rating scale: 0–4 pts—no disability; 5–14 pts— slight disability; 15–24 pts—moderate disability; 25–34 pts—severe disability; more than 35 pts—total disability. Percentage interpretation of the ODI: 10–20%—slight disability, 21–40%—mild disability, 41–60%—moderate disability, and 61–80%—severe disability [23]. The Cronbach’s alpha coefficient value for the SF-36 ranges from 0.71 to 0.87 [24].

#### 2.4.3. Quality of Life

The SF-36 is a tool for the overall assessment of HRQOL. It is made up of 36 questions that comprise eight domains relating to mental and physical health. These domains consist of physical functioning, social functioning, role limitations due to physical problems, role limitations due to emotional problems, mental health, general health perception, bodily pain, and vitality. The questionnaire is employed to assess health in various medical and psychosocial categories. The individual questions relate to (1) physical functioning (PF)—10 questions, (2) role limitations due to physical problems (RP)—4 questions, (3) bodily pain (BP)—2 questions, (4) general health perception (GH)—5 questions, (5) vitality (VT)—4 questions, (6) social functioning (SF)—2 questions, (7) mental health (MH)—5 questions, (8) role limitations due to emotional problems (RE)—3 questions. Each answer in each domain is assigned a corresponding number of points. The score ranges from 0 to 100 in each category. The lower the score, the worse the QOL [25]. The Cronbach’s alpha coefficient value for the SF-36 is very high and ranges from 0.83 to even 0.95 [26].

### 2.5. Ethical Considerations

The study design was approved by the Bioethics Committee of the Wroclaw Medical University (No. KB-136/2014). The study was conducted following the principles of Good Clinical Practice (GCP), ensuring high quality, reliability, and data safety. The provisions of the Declaration of Helsinki (DoH) on patient rights were followed throughout the study in accordance with ethical and regulatory requirements. The study followed the guidelines for the presentation of non-randomized studies (NRSs) [27].

### 2.6. Sample Size

The study employed a meticulous approach to determine the sample size using Statistica 12.0 (TIBCO, Software Inc., Palo Alto, CA, USA). Based on a priori considerations and clinical insights, an estimated medium effect size of 0.5 for pain reduction, disability improvement, and quality-of-life enhancement was anticipated with the surgical intervention at three measurement time-points: before surgery, 24 h after surgery, and 3 months after surgery. The significance level was set at 0.05, signifying a 5% probability of a type I error, while a power of 0.80 was targeted to achieve an 80% likelihood of identifying a genuine effect, thereby minimizing the risk of a type II error. The use of a two-tailed test facilitated the detection of any significant difference, whether manifested as an increase or decrease in pain. Consequently, the determined total sample size of 96 individuals was considered essential for the statistical precision and reliability of the study.

### 2.7. Statistical Analysis

Statistical analysis was performed using Statistica 12.0 (TIBCO, Software Inc., Palo Alto, CA, USA). Arithmetic means, medians, standard deviations, and range of variation (extreme values) were calculated for quantitative variables. For qualitative variables, their frequencies of occurrence (percentages) were calculated. All quantitative variables examined were tested using the Shapiro–Wilk test to determine the type of distribution. Comparisons of VAS scores before surgery, 24 h after surgery, and 3 months after surgery were made using a one-way analysis of variance (ANOVA) and post hoc test (Tukey’s test). Comparisons of scores before and three months after surgery were performed using the parametric Student’s *t*-test for dependent samples or the non-parametric Wilcoxon test, depending on whether the test assumptions were met. Comparisons of qualitative variables before and three months after surgery were performed using the chi-squared test (χ^2^). An α = 0.05 was used for all comparisons, and the resulting *p* values were rounded to four decimal places. In addition, the relationship between the selected variables was determined using the Pearson correlation test (α = 0.05).

## 3. Results

### 3.1. Patients’ Characteristics

A total of 128 patients took part in the first stage of the study before surgery. Due to the failure of 18 participants to register for the second stage of the study within three months after surgery, the complete results of 110 subjects were included in the final statistical analysis of the collected material. Table 1 shows the detailed characteristics of the study group (n = 110). Background information was included, i.e., age, height and weight, BMI, sex, marital status, place of residence, and education.

Furthermore, background information on professional activity was obtained. The largest proportion of individuals among the surveyed group indicated “salary” as a source of income (63.64%, n = 70). This was followed by “benefits” (63.64%, n = 70), “disability pension” (9.09%, n = 10), “pension” (8.18%, n = 9), “no source of income” (4.55%, n = 5), and “parents’ dependent” (2.73%, n = 3). More than 70% (n = 77) of subjects were blue-collar workers before the surgery, 23.85% (n = 26) were white-collar workers, and 5.50% (n = 6) never worked. Respondents also specified their position at work. The largest percentage of subjects declared they were constantly on the move (52.34%, n = 56), with a large proportion doing sedentary work (30.84%, n = 33).

More than 50% of participants related their complaints to work (59.09%, n = 65), and the remainder to sports, household chores, or other factors. Moreover, 67.27% (n = 74) of subjects declared no family history of disc disease. In 110 subjects (100.0%), the disc disease involved the L3–L5 lumbar region. The majority of the subjects’ complaints lasted between 5 and 10 years (41.82%, n = 46). More than 95% of participants reported experiencing pain associated with discopathy, and in most cases, this was chronic (85.45%, n = 94). One in four participants declared a sedentary lifestyle (25.45%, n = 28), and more than half reported little physical activity (52.73%, n = 58). In addition, 32.73% (n = 36) of the study participants gardened and 20.91% played sports (n = 23).

### 3.2. Pain Intensity

**Figure 1:**
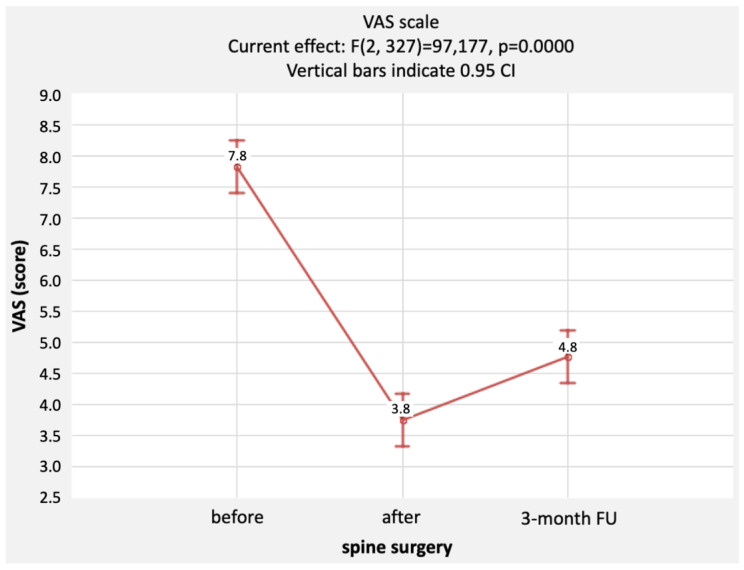
Comparison of ODI questionnaire scores before and 3 months after surgery [pts], together with a representation of the distribution of scores. Notes: mean, standard error; 1.96 * standard error.

The VAS was employed to assess pain complaints. Assessments were made before surgery, 24 h after surgery, and 3 months after surgery. The mean pain intensity before surgery was 7.8 pts (min.–max.: 0–10 pts, SD = 2.3 pts) and decreased significantly 24 h after surgery, with a mean score of 3.8 pts (min.–max.: 0–10 pts, SD = 2.4 pts). After three months, pain intensity increased, but the result was still significantly better than before surgery. A mean score of 4.8 pts (min.–max.: 0–10 pts, SD = 2.4 pts) was found. The scores were statistically significantly different (*p* = 0.0000). The post hoc analysis is shown in Table 2, and Figure 1 additionally shows the 95% confidence interval (CI) for the values obtained at each assessment stage.

### 3.3. Disability Level

Patients’ disability level scores using the ODI are shown as points (Table 3), percentages (Table 4), and categories: I—0–20% no disability; II—21–40% slight disability; III—41–60% moderate disability; IV—61–80% severe disability; V—81–100% total disability (Table 5). 

**Figure 2:**
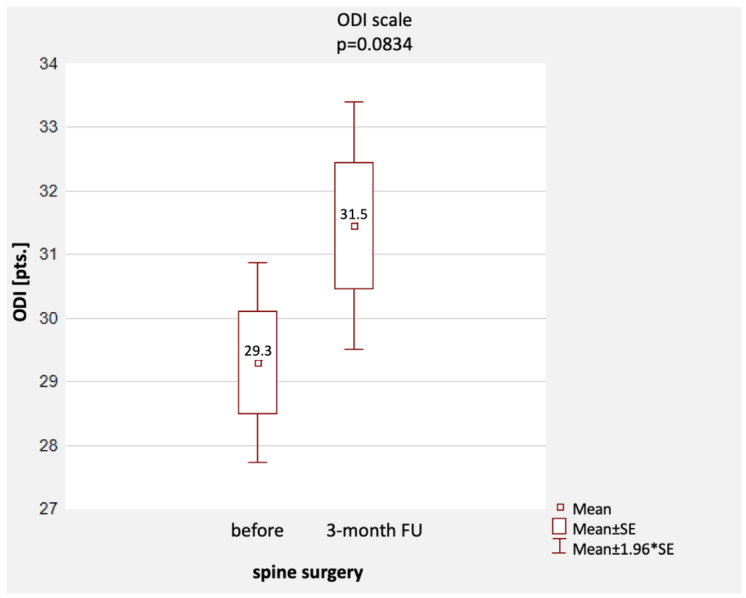
Comparison of VAS scores before surgery, 24 h after surgery, and 3 months after surgery. Notes: determination of 95% confidence intervals was included.

The mean ODI score [pts] before surgery was 29.3 pts (min.–max.: 11–49 pts, SD = 8.4 pts, i.e., slight disability), and three months after surgery, it increased slightly to a mean value of 31.5 pts (min.–max.: 10–55 pts, SD = 10.4 pts, i.e., patients in the study group still had a slight disability). The scores were not statistically significantly different (*p* = 0.0834) (Table 3 and Figure 2). 

**Figure 3:**
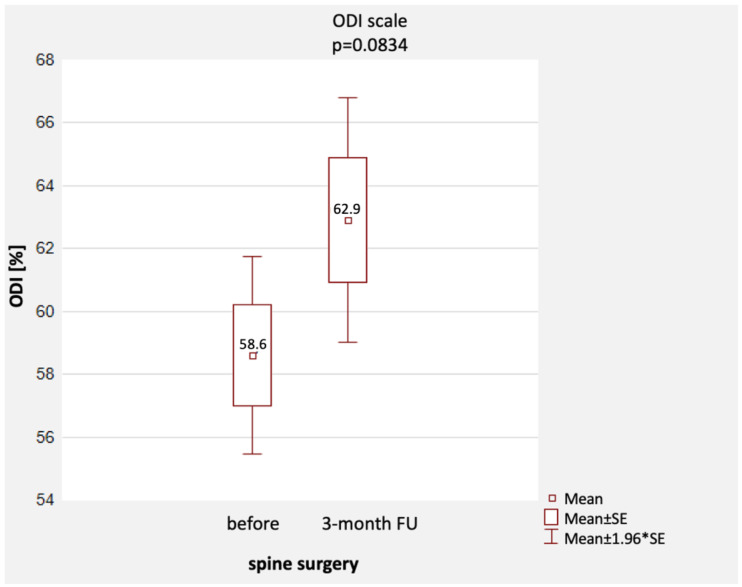
Comparison of ODI scores before and 3 months after surgery [%], together with a representation of the distribution of scores. Notes: mean, standard error; 1.96 * standard error.

The mean ODI score [%] before surgery was 58.6% (min.–max.: 22–98%, SD = 16.8%), and three months after surgery, it increased slightly to a mean value of 63.0% (min.–max.: 20–100%, SD = 20.8%). The scores were not statistically significantly different (*p* = 0.0834) (Table 4 and Figure 3). 

A comparison of the ODI scores before and three months after surgery by category is shown in Table 5. There were no statistically significant differences between the scores. 

### 3.4. Quality of Life

There was a statistically significant increase in scores (QOL improvement) at three months after surgery in the following domains: PF (increase in mean values by 8.7%; *p* = 0.0176), BP (increase in mean values by 26.2%; *p* = 0.0000), VT (increase in mean values by 5.8%; *p* = 0.0132) and MH (increase in mean values by 6.2%; *p* = 0.0163), and a decrease (worsening of QOL) in RP (decrease in mean values by 3.8; *p* = 0.0013) and GH (decrease in mean values by 6.7% (*p* = 0.0112) (Table 6). 

## 4. Discussion

The VAS is often used in studies on pain intensity and QOL before and after intervertebral disc surgery for various indications. It is a tool that serves an auxiliary function to assess lumbosacral pain intensity [28]. In the study material, the assessment was performed three times: before surgery, shortly after surgery (after 24 h), and three months after surgery. Before surgery, the mean score was 7.8 points on a 10-point scale, while after surgery, the index dropped to 3.8 points, increasing again to 4.8 points three months after surgery. The increase in the VAS score and thus in pain intensity in the long term compared to its value immediately after surgery can most likely be explained by the patients’ intensive intake of painkillers in the postoperative period. In the long term, after surgery, the intensity of pharmacotherapy for pain decreases, and patients often return to their previous work, so their pain perception is higher than immediately after surgery. Still, it does not reach the average value before surgical treatment. This shows that in this group of patients, the surgery fulfilled its role in terms of pain control, as it contributed to a significant reduction in pain (*p* = 0.0000).

Our results correspond with those of other authors investigating the intensity of pain associated with intervertebral disc disease using the VAS. The follow-up and information collection period varies, making it difficult to directly compare these results. Puolakka et al. [29] conducted a study to assess risk factors for absenteeism due to back pain in patients after lumbosacral surgery. In those patients, the follow-up period was five years after surgery. Two months after surgery, data on pain intensity were collected using the VAS. The results, illustrated by the coefficient in relation to baseline (preoperative) intensity, show a significant reduction in pain intensity (coefficient: 0.58, confidence interval: 0.38–0.79; *p* < 0.001) for limb pain assessment but no significant change for spinal pain (*p* = 0.15). The mean pain intensity in the presented population before surgery (VAS = 7.4; 95% CI: 5.7–8.9) was comparable to the results of this study. On the other hand, Solberg et al. [30] analyzed the concerns of patients with disc disease about the deterioration of their condition after surgery. The follow-up period in that case was 12 months, and after that time, data on self-assessed pain were collected again using the VAS. For lumbosacral pain, the intensity decreased from 5.17 to 2.13 (*p* < 0.001), while for limb pain, it dropped from 6.34 to 1.68 (*p* < 0.001). The decrease in pain was significant and noticeable in both assessments. 

The disability level of the patients studied was evaluated using the Oswestry Disability Index (ODI). Scores were presented as points, percentages, and categories of disability according to Rąpała, where the highest level (V) indicates total disability. As in the other cases, data were collected before surgery and three months after surgery. For comparative purposes, reporting the scores in percentage form is most relevant. In the study material, the mean ODI was 58.6 ± 16.8 pts before surgery, while three months after surgery, it increased to 62.9 ± 20.8 pts. The score did not reach statistical significance (*p* = 0.0834); i.e., the patients’ disability level did not change significantly three months after surgery.

Those findings differ from the data reported by other authors, where the mean score of the patients according to the ODI was 56.29 ± 16.77 before surgery and 37.08 ± 19.33 after surgery (*p* < 0.001), suggesting a significant improvement. The source of this discrepancy is unclear, especially as the preoperative scores were comparable (58.6 vs. 56.29). Perhaps they result from individual differences between the patient groups (age, sex), their worse condition before the surgery itself, or different postoperative care. Furthermore, some authors suggest that the three-month follow-up period is too short and should be extended to at least one year [29,30], while others believe that as little as three months is sufficient and any results of studies performed during that period show a significant correlation with prognosis and long-term outcomes [31].

An improvement in mobility according to the ODI was further observed in the study by Häkkinen et al. [32] one year after surgery—the mean ODI score decreased by 38% in women and by 24% in men (*p* < 0.001). Notably, a significant proportion of that improvement occurred already in the first six weeks (88% in women and 80% in men) after surgery, implying that the recovery period was relatively rapid in that population. A reduction in the disability level after surgery, according to the ODI, was also reported by other authors [33,34], reaching the statistical significance of the results presented. Similar values were also reported by Dewing et al. [35], who found that a reduction of more than 25% was recorded in as many as 81% of patients enrolled in their study (before surgery—53.55, after surgery—21.22), as well as Solberg et al. [30] (analogous values: 49.5 and 13.4). The finding of Dewing et al. [35] is particularly striking, but it should be mentioned that in his case, the study population consisted mainly of young people (mean age: 27 years), whereas in this presented material, the mean age was 44.8 years. This implies that age may play an important role in prognosis and the improvement in life observed by patients after surgery.

In connection with the analysis of the ODI, many authors propose that a reduction in its average value by a certain number of percentage points be considered a significant improvement in mobility. Many authors consider a 10% reduction in the ODI score as significant, while some adopt even lower values, around 8% [36,37]. Regardless of the values adopted, it should be noted that the ODI score fluctuates somewhat over time, as found, for example, by Häkkinen et al. [32]. The authors examined various aspects of QOL at two and 14 months after surgery and noted no significant differences between these measurements.

In our study, we analyzed the scores of the SF-36 before surgery and three months after surgery. All eight domains of the scale were compared. A significant increase in the scores obtained, which is equivalent to an improvement in QOL, was obtained in the following components: PF (physical functioning)—an increase by 8.7% (*p* = 0.0176); BP (bodily pain)—an increase by 26.2% (*p* = 0.0000); VT (vitality)—an increase by 5.8% (*p* = 0.0132); MH (mental health assessment)—an increase by 6.2% (*p* = 0.0163); PH (mental health)—an increase by 11.1% (*p* = 0.0057); and FH (physical health)—an increase by 9.4% (*p* = 0.0017). Decreases in the scale’s mean values, signifying a deterioration in QOL, were obtained for the RP (role limitations due to physical problems—a decrease by 3.8% (*p* = 0.0013) and GH (general health perception—a decrease by 6.7% (*p* = 0.0112) domains.

The study found that most components of QOL improved in the operated patients. In particular, the reduction in pain perception appears to be significant. It reached the highest value, resulting in the highest percentage increase in the corresponding component (BP). It follows that pain plays a prominent role in patients’ perception of well-being, and its reduction or elimination is a fundamental issue. Reductions in QOL were noted in the RP and GH components, presumably due to incomplete recovery and rehabilitation (three months after surgery may be too short a time for many patients), affecting poorer self-assessment of overall physical health and limitations in performing daily activities. This could indicate the need for increased care and rehabilitation in those patients, which would contribute to their faster recovery.

Previous studies of patients after surgery for intervertebral disc disease report significant improvements in all components of QOL according to the SF-36 [38,39]. The greatest improvement, analogously to this study, is observed in the GB domain, and only the GH domain did not improve significantly, which is consistent with the findings of this study. Moreover, patients’ physical and mental health scores recorded significant improvements at six and 12 months after lumbar discectomy [40]. The study indicates that a 10% increase in any component of the SF-36 should be considered a significant change (improvement) in clinical outcomes, which suggests an improvement in patients’ lives. Studies evaluating the QOL of patients with lumbosacral pain and sciatica symptoms had significantly lower scores of individual domains of the SF-36 in relation to population averages, as well as in terms of pain (VAS), level of disability (ODI) and QOL (EQ-5D-5L) [41]. A study by Farzanegan et al. [40] presents physical and mental health scores on the SF-36, which improved significantly at 6 and 12 months after lumbar discectomy. The mean improvement in physical health scores was significantly higher in women than in men, with no significant sex differences in mental health. Finally, Youn et al. [42] report that VAS, ODI, and SF-36 scores improved significantly at the one-month follow-up visit compared to average baseline values and were maintained over the two-year follow-up period after endoscopic partial facetectomy (EPF). Correlation analysis showed significant correlations between various preoperative factors and clinical outcomes.

All of the aforementioned facts show that surgery in the vast majority of patients induces a noticeable improvement in QOL in almost all aspects. However, individual domains may undergo differential increases. This study also confirms those findings.

### Strengths and Limitations

The present study has several strengths. The research explores an important and topical issue of the evaluation of patients undergoing lumbar disc surgery. The inclusion of patients from both elective and emergency admissions broadens the generalizability of the findings. The study included a substantial number of participants, providing a robust dataset for analysis and employing standardized self-reported assessments, enhancing the reliability and comparability of the collected data.

There are also potential methodological limitations. The study was conducted at a single medical center, which may limit the generalizability of the findings to other healthcare settings. The study was conducted in one region of Wroclaw (Poland), and the findings may not be applicable to regions with different healthcare systems or patient populations. In future studies, it is advisable to complement self-reported assessments with objective measures to provide a more comprehensive evaluation of the subject matter.

## 5. Conclusions

The surgical procedure played an important role in improving the QOL of patients operated on for lumbar disc disease. The surgical procedure effectively reduced pain intensity and severity, and the reduction was most noticeable 24 h after surgery. The patients’ disability level did not significantly decrease three months after surgery, indicating that the surgery did not affect the patients’ mobility. The practical applications of this research include guiding treatment decisions for lumbar disc surgery, helping patients understand post-surgery outcomes, optimizing healthcare resource allocation, prioritizing future research, and informing healthcare policies for better patient care, ultimately improving the lives of patients undergoing lumbar disc surgery.

## Figures and Tables

**Table 1:** Characteristics of the study group.

Characteristics of the Group		
Age [years]	x	44.8
	min.–max.	18.0–76.0
	SD	13.1
Body height [cm]	x	170.6
	min.–max.	152.0–193.0
	SD	8.8
Body weight [kg]	x	76.6
	min.–max.	44.0–120.0
	SD	16
BMI [kg/m^2^]	x	26.2
	min.–max.	17.6–39.1
	SD	4.5
LBP period [months]	x	48.4
	min.–max.	6.8–121.0
	SD	49.2
Sex	female	50.91%
		n = 56
	male	49.09%
		n = 54
Marital status	married	63.64%
		n = 70
	widowed	3.64%
		n = 4
	single	32.72%
		n = 36
Place of residence	urban	73.64%
		n = 81
	rural	26.36%
		n = 29
Education	primary	13.64%
		n = 15
	vocational	33.64%
		n = 37
	secondary	39.09%
		n = 43
	higher or higher vocational	13.64%
		n = 15

Abbreviations: n, number of participants; x, mean; min., minimum value; max., maximum value; SD, standard deviation; cm, centimeters; kg, kilograms; m^2^, square meters.

**Table 2:** Comparison of VAS scores before surgery, 24 h after surgery, and 3 months after surgery.

		n	x	Me	Min.	Max.	SD
VAS [pts]	Before	110	7.8	8.0	0.0	10.0	2.3
	After	110	3.8	3.5	0.0	10.0	2.4
	After 3 months	110	4.8	5.0	0.0	10.0	2.1
*p*-value (main effect)		*p* = 0.0000 *					
*p*-value (multiple comparisons)		Before vs. after—*p* = 0.0000 ** Before vs. after 3 months—*p* = 0.0000 ** After vs. after 3 months—*p* = 0.0024 **					

Abbreviations: n, number of participants; x, mean; Me, median; min., minimum value; max., maximum value; SD, standard deviation; pts, points; vs., versus; VAS, Visual Analogue Scale. Notes: * ANOVA, ** Tukey’s test.

**Table 3:** Comparison of ODI scores before and 3 months after surgery [pts].

		n	x	Me	Min.	Max.	SD
ODI	Before	110	29.3	28.0	11.0	49.0	8.4
	After 3 months	110	31.5	31.5	10.0	55.0	10.4
*p*-value		*p* = 0.0834 *					

Abbreviations: n, number of participants; x, mean; Me, median; min., minimum value; max., maximum value; SD, standard deviation; pts, points; ODI, Oswestry Disability Index. Notes: * Student’s *t*-test.

**Table 4:** Comparison of ODI scores before and 3 months after surgery [%].

		n	x	Me	Min.	Max.	SD
ODI %	Before	110	58.6	56.0	22.0	98.0	16.8
	After 3 months	110	62.9	63.0	20.0	100.0	20.8
*p*-value		*p* = 0.0834 *					

Abbreviations: n, number of participants; x, mean; Me, median; min., minimum value; max., maximum value; SD, standard deviation; ODI, Oswestry Disability Index. Notes: * Student’s *t*-test.

**Table 5:** Comparison of ODI scores before and 3 months after surgery by category.

		Before Surgery	3 Months after Surgery	*p*-Value *
No disability	n	0	3	*p* = 0.1096
	%	0.00%	2.73%	
Slight disability	n	12	15	
	%	10.91%	13.64%	
Moderate disability	n	49	35	
	%	44.55%	31.82%	
Severe disability	n	36	36	
	%	32.73%	32.73%	
Total disability	n	13	21	
	%	11.82%	19.09%	

Abbreviations: n, number of participants; %, percentage. Notes: * χ^2^ test.

**Table 6:** Comparison of SF-36 component scores before and 3 months after surgery.

		x	Me	Min.	Max.	SD	*p*-Value
PF [%]	Before	36.6	35.0	0.0	95.0	23.6	*p* = 0.0176 **
	After 3 months	45.3	45.0	0.0	100.0	25.6	
RP [%]	Before	22.6	25.0	0.0	43.8	6.6	*p* = 0.0013 *
	After 3 months	18.8	25.0	0.0	31.3	9.4	
BP [%]	Before	12.0	0.0	100.0	17.7	12.0	*p* = 0.0000 **
	After 3 months	38.2	42.0	0.0	100.0	21.6	
GH [%]	Before	44.3	45.0	5.0	92.0	17.1	*p* = 0.0112 **
	After 3 months	37.6	35.0	0.0	100.0	20.3	
VT [%]	Before	49.8	50.0	12.5	93.8	17.2	*p* = 0.0132 **
	After 3 months	55.6	56.3	6.3	87.5	15.3	
SF [%]	Before	53.2	50.0	12.5	100.0	15.7	*p* = 0.8089 **
	After 3 months	52.6	50.0	12.5	75.0	12.0	
RE [%]	Before	14.1	16.7	0.0	41.7	11.9	*p* = 0.9214 **
	After 3 months	14.2	16.7	0.0	25.0	11.6	
MH [%]	Before	48.6	50.0	10.0	90.0	17.2	*p* = 0.0131 **
	After 3 months	54.8	52.5	20.0	90.0	15.5	

Abbreviations: x, mean; Me, median; min., minimum value; max., maximum value; SD, standard deviation; %, percentage; PF, physical functioning; RP, role limitations due to physical problems; BP, bodily pain; GH, general health perception; VT, vitality; SF, social functioning; RE, role limitations due to emotional problems; MH, mental health assessment. Notes: * Student’s *t*-test; ** Wilcoxon test.

## Data Availability

The authors confirm that all data underlying the findings described in this manuscript are available in full to all interested researchers upon request.
